# Interlaboratory comparison of immunoprecipitation-based testing for myositis-specific autoantibodies

**DOI:** 10.3389/fimmu.2026.1918595

**Published:** 2026-09-28

**Authors:** Kimberly Loh, Georgina R Harvey, Xavier Bossuyt, Anna Brusch, Chris Bundell, Mei Cheng, Masataka Kuwana, Hui Lu, Birthe Michiels, Johan Ronnelid, Ira N Targoff, Akira Yoshida, Neil J McHugh, Sarah L Tansley

**Affiliations:** 1 Department of Life Sciences, University of Bath, Bath, United Kingdom; 2 Department of Laboratory Medicine, University Hospitals Leuven, Leuven, Belgium and Department of Microbiology, Immunology and Transplantation, KU Leuven, Leuven, Belgium; 3 Department of Clinical Immunology, PathWest Laboratory Medicine, Perth, WA, Australia; 4 Arthritis & Clinical Immunology Research Program, Oklahoma Medical Research Foundation, Oklahoma City, OK, United States; 5 Department of Allergy and Rheumatology, Nippon Medical School Graduate School of Medicine, Tokyo, Japan; 6 Department of Immunology, Genetics and Pathology, Uppsala Universitet, Uppsala, Sweden

**Keywords:** autoantibodies, idiopathic inflammatory myopathies, immunoprecipitation, interlaboratory variability, myositis

## Abstract

**Introduction:**

Myositis-specific autoantibodies (MSAs) are clinically important biomarkers that help classify idiopathic inflammatory myopathies and guide clinical management, but reliable detection remains challenging. Immunoprecipitation (IP) is widely regarded as a reference method for MSA detection in specialist laboratories. However, IP comprises a range of related techniques rather than a single standardised assay, and variability between expert laboratories using different IP-based workflows is not well characterised.

**Methods:**

Serum samples previously submitted to the Bath Diagnostic Serology Service were distributed to three collaborating reference laboratories. Centres analysed samples using their routine workflows, which included radiolabelled protein IP, RNA-IP, IP-western blotting, IP-mass spectrometry, and supplementary immunoassays. Results were interpreted within each laboratory's diagnostic framework, and interlaboratory concordance of MSA detection and specificity assignment was evaluated.

**Results:**

Thirty-two samples were included. Overall concordance across overlap groups ranged from 63–78%. Agreement varied by MSA specificity and methodological scope. Anti-Zo, anti-NXP2 and anti-TIF1γ demonstrated the highest reproducibility, whereas anti-PL-7, anti-Jo-1, anti-EJ, anti-Ha and anti-HMGCR were identified only by isolated laboratories. Differences were consistent with variation in IP method, including antigen source, detection modality and availability of reference sera. Complementary assays played an essential role in anti-MDA5, anti-HMGCR and anti-NXP2 antibody detection.

**Discussion:**

These findings demonstrate that IP-based testing should be considered a framework of expert diagnostic practice rather than a single uniform assay. Greater transparency in laboratory methods and reporting practices may improve interpretation of autoantibody results and support more consistent comparison of MSA results across clinical and research settings.

## 1 Introduction

Idiopathic inflammatory myopathies (IIM) are heterogeneous, acquired autoimmune disorders characterised by muscle inflammation and multisystem involvement (1). The classification of these diseases continues to evolve, but currently recognised subtypes include dermatomyositis (DM), polymyositis (PM), anti-synthetase syndrome (ASyS), overlap myositis (OM), inclusion body myositis (IBM), and immune-mediated necrotising myopathy (IMNM) (2). Over the past two decades, advances in the identification of myositis-specific autoantibodies (MSAs) have significantly improved understanding of disease heterogeneity and contributed to more refined clinical classification of myositis. With the availability of appropriate testing methods, approximately 40–89% of patients with IIM are now recognised to harbour an identifiable MSA (3–6).

MSAs are particularly valuable biomarkers because individual antibody specificities are strongly associated with distinct clinical phenotypes (7). These autoantibodies are typically mutually exclusive, with most patients expressing a single MSA specificity. As a result, they define relatively homogeneous clinical subsets within the otherwise heterogeneous spectrum of IIM. Anti-Mi-2, anti-TIF1γ, anti-NXP2, anti-MDA5 and anti-SAE autoantibodies are associated predominantly with DM; antibodies directed against aminoacyl-tRNA synthetases (ARS), including Jo-1, PL-7, PL-12, EJ, OJ, KS, Zo and Ha, are associated with ASyS; and anti-SRP and anti-HMGCR autoantibodies are associated with IMNM. Certain MSAs are also associated with clinically important complications. Anti-ARS antibodies are frequently associated with interstitial lung disease (ILD), while anti-TIF1γ antibody positivity is associated with an increased risk of malignancy in adult-onset disease (7). Accurate identification of these antibodies is therefore important for diagnostic evaluation, risk stratification, and guiding clinical management.

A variety of laboratory techniques are available for MSA detection. In routine clinical practice, testing is most commonly performed using commercially available immunoassays in hospital and private laboratories. Many of these assays employ multiplex formats that allow simultaneous detection of several autoantibody specificities. However, the repertoire of autoantigens included within these assays varies between platforms, and differences in sensitivity and specificity have been reported for certain MSAs (8, 9). As a result, clinically relevant autoantibodies may be missed or inconsistently detected depending on the assay used.

Immunoprecipitation (IP) using cell extracts has historically played a central role in the discovery and characterisation of MSAs and is widely regarded as the ‘gold standard’ method for MSA detection in specialist laboratories (7). Unlike many targeted commercial assays, IP enables detection of autoantibodies recognising conformational epitopes of native proteins and multiprotein complexes and can reveal previously uncharacterised immunoreactivities that may represent novel autoantibody specificities (10). Despite being widely referred to as a reference method, IP is not a single standardised assay but rather, it encompasses a range of related IP-based laboratory techniques that differ in (i) the selection of antibody immobilisation matrix and antigen source, (ii) the methods used to recover and prepare the immunoprecipitated proteins and/or RNPs for subsequent analysis, and (iii) the downstream approaches used to separate, detect and identify the immunoprecipitated proteins and/or RNAs. While such approaches share a common IP step, they differ in how the immunoprecipitated material is subsequently detected and identified. In protein-based IP approaches, patient serum is incubated with a cell extract, allowing autoantibodies to bind to their native target proteins or protein complexes. The resulting immune complexes are captured for subsequent analysis. For example, expert laboratories specialising in the detection of autoantibodies may employ ^35^S-methionine-radiolabelled cell extracts during radioimmunoprecipitation of proteins (^35^S-IP), with immunoprecipitated proteins separated by SDS-PAGE and detected according to characteristic electrophoretic patterns. RNA-IP may be used to identify autoantigenic ribonucleoprotein (RNP) complexes via detection of RNA species on urea-PAGE gels. Alternatively, IP may be combined with a variety of downstream techniques such as Western blotting (IP-WB), which combines IP with antigen-specific WB, or mass spectrometry (IP-MS), which identifies immunoprecipitated proteins and associated complexes by mass spectrometry (8, 11).

In practice, IP assays are often locally developed and implemented within individual laboratories, reflecting available infrastructure, technical expertise and complementary and/or confirmatory testing strategies. Consequently, protocols may differ substantially between centres. These methodological differences can influence which autoantigens are detected and how results are interpreted. Perhaps no single form of IP can detect the full spectrum of known MSAs, and variation in locally implemented IP-based workflows may therefore contribute to differences in reported results between laboratories. Importantly, such variability may reflect the absence of standardised IP-based assay systems rather than limitations inherent to IP technology itself.

Despite the important role of IP techniques in MSA testing, relatively little information is available regarding the extent of inter-laboratory variability when different IP-based workflows are applied to the same samples. Most previous studies have compared commercial immunoassays with IP results obtained from a single reference laboratory rather than examining concordance across multiple expert centres (12–14). Furthermore, in routine diagnostic practice the final antibody assignment often reflects integration of results from several complementary assays rather than the outcome of a single IP-based test.

In this study, we compared MSA results obtained from shared serum samples tested across multiple international reference laboratories, each employing different IP-based workflows. The aim was to evaluate inter-laboratory variability in MSA detection across expert centres and to examine patterns of concordance and divergence in reported MSA specificities, with particular attention to the methodological factors that may contribute to these differences.

## 2 Methods

### 2.1 Study design and sample distribution

Patient sera used for this study had previously been submitted to the Bath Diagnostic Serology Service and were retained as anonymised residual samples for quality-control purposes. In addition, two plasmapheresis samples donated by the PathWest Laboratory Medicine, Perth, Australia for the purposes of this study, were used. The Australian samples were from patients with confirmed myositis. Remaining samples had been submitted with a request for extended spectrum autoantibody testing by IP but no clinical or demographic information was available. These samples included cases likely to be diagnostically challenging or to contain difficult-to-detect MSAs. In practice, due to limited availability of such samples, selection was based on sera demonstrating features suggestive of potential MSA reactivity, including cytoplasmic immunofluorescence patterns, borderline or discordant results on line immunoassay, or uncertain findings on prior IP testing. Allocation to participating laboratories was based on availability and logistical considerations and was not determined by the known detection capabilities or reported specificities of individual laboratory workflows.

Multiple aliquots of each serum sample were prepared at the University of Bath and distributed to the three collaborating expert reference laboratories in Tokyo, Japan; Oklahoma, USA; and Leuven, Belgium for independent analysis using each laboratory’s routine diagnostic workflow.

Due to logistical constraints such as differences in availability of individual sera, not all centres received identical sets of samples. However, partial overlap between laboratories enabled inter-laboratory comparisons across groups of samples analysed by two, three, or four laboratories.

To maintain methodological comparability across centres, samples for which no IP-based testing had been performed and only non-IP assay results were available were excluded from the primary concordance analyses. Samples in which IP-based testing had been performed were included irrespective of result, including where a final MSA specificity was identified only by a complementary non-IP assay.

All samples were anonymised prior to distribution. The use of residual diagnostic sera for method comparison and assay evaluation was conducted in accordance with institutional guidelines and was deemed exempt from formal ethical review given that this was a quality control exercise.

### 2.2 Laboratory methodologies

All participating centres employed IP-based approaches as part of their routine diagnostic workflows, although the specific methodologies differed between laboratories. The coordinating laboratory at Bath performed ^35^S-IP using K562 cell extracts as the primary method, as previously described (9). Where a 140 kDa band was detected following subsequent autoradiography of SDS-PAGE gels, reflex in-house ELISA testing for anti-MDA5 and anti-NXP2 was performed to assist in antigen specificity assignments.

The Tokyo laboratory performed RNA-IP using HeLa cell extracts for the detection of a number of MSAs which recognise particular RNP autoantigens, alongside IP-WB, targeting selected myositis autoantigens including Mi-2, TIF1γ, NXP2, SAE and Ku. In addition, commercially available ELISA (MESACUP™; Medical and Biological Laboratories, Tokyo, Japan) was performed for the detection of anti-MDA5, TIF1γ, Mi-2, SRP, HMGCR, SAE, NXP2, and anti-ARS antibodies. The anti-ARS ELISA detects antibodies to Jo-1, PL-7, PL-12, EJ and KS but does not differentiate between individual synthetase specificities. Individual anti-ARS specificities were therefore assigned using RNA-IP in conjunction with available reference sera. The performance characteristics of these ELISA assays have been described recently (15). Where the same MSA specificity was assessed by more than one method, a specificity identified by either method was included in the final MSA result. IP-WB assays for systemic sclerosis-associated autoantigens (Th/To, PM-Scl, Ku, U11/U12RNP and U3RNP) were also performed but were outside the scope of this analysis. Similar RNA-IP and IP-based workflows have been described previously (16).

The Leuven laboratory utilised IP-MS to identify precipitated proteins and associated complexes, as previously described (17, 18). The assay is validated for the detection of antibodies to eIF2B, Zo, OJ, KS, PL-7, PL-12, EJ (cytoplasmic targets) and Th/To, fibrillarin-U3 RNP, PM-Scl, TFIID, RuvBL1/2, TIF1β, TIF1γ, Ku, Mi-2, THO complex (nuclear targets). HEp-2 indirect immunofluorescence (IIF) was performed and used to guide selection of HeLa cell extract for IP-MS, and the reported patterns were included, where available. Where a nuclear IIF pattern was detected on Hep-2-cell slides, a nuclear-enriched HeLa cell extract was used for IP-MS; where cytoplasmic staining was observed, a cytoplasmic extract was used. Additionally, both extracts were used in some samples showing both nuclear and cytoplasmic patterns. For IIF-negative samples, a nuclear extract was used. A commercial multi-antigen dot immunoassay (MYOS12DIV-24; D-Tek, Mons, Belgium) was selectively performed as a complementary assay within the laboratory’s diagnostic workflow and was not applied to all samples. The panel included anti-MDA5, NXP2, TIF1γ, Jo-1, PL-7, PL-12, EJ, SRP, Mi-2, Ro52, SAE1 and SAE2.

The Oklahoma laboratory employed three IP-based approaches, including RNA-IP and ^35^S-IP, both using HeLa cell extracts. Additional, complementary IP-WB using K562 cell extracts was performed for the detection of MSAs recognising selected autoantigens, specifically TIF1γ, NXP2 and MDA5.

Although IP-based methods formed the core of testing workflows across all four laboratories, the workflows differed in their cell and extract sources, the downstream methods used to detect or identify the immunoprecipitated material, and the complementary assays and reference materials used to assign antibody specificity. Detailed technical protocols for the individual methods are provided in the cited studies (9, 14–17). The reported MSA specificity therefore reflected each laboratory’s integrated diagnostic interpretation rather than the result of a single assay. Participating centres provided assay-level outputs generated within their routine workflows, which were shared centrally with the coordinating laboratory in Bath. The final MSA specificity for each sample based on the combined results from that centre is shown in **Tables 1**–**3**. Where specificity assignment relied primarily on a supplementary assay rather than on an IP-based assay result, this was noted in the analysis.

**Table 1:** Laboratory-reported MSA and MAA specificities for samples analysed by all four centres.

Sample	IIF	Bath	Tokyo	Leuven	Oklahoma
S1	nuclear fine speckled and nucleolar staining	─ *PM-Scl*	SRP* Th/To, Sm	† *PM-Scl*	SRP *PM-Scl*
S2	negative	NXP2	NXP2	NXP2*	NXP2 *Ro (weak)*
S3	nuclear fine speckled and cytoplasmic dense fine speckled	Zo	† SS-B, Ro52*	Zo *Ro60*	Zo *Ro*
S4	nuclear fine speckled	TIF1γ	TIF1γ	TIF1γ	TIF1γ
S5	nuclear fine speckled and nucleolar staining + cytoplasmic dense fine speckled	─	─	─	─
S6	nuclear fine speckled	─	─	─	EJ (weak)
S7	dense fine speckled	†	HMGCR*	† *PSIP1/DFS70*	†

*Positive result obtained using a complementary assay (ELISA or dot immunoassay), rather than the IP-based assay; this includes specificities outside the detection scope of the IP method used.

MAAs in italics.

Green: MSA specificity reported by the laboratory.

Tan: No MSA reported.

Blue (†): MSA specificity outside the reporting scope of the laboratory workflow for this study.

**Table 2:** Laboratory-reported MSA and MAA specificities for samples analysed by three centres.

Sample	IIF	Bath	Tokyo	Leuven	Oklahoma
S8	nuclear fine speckled	MDA5	MDA5*	MDA5*	
S9	nuclear fine speckled	─	─	─	
S10	negative	─	─	─	
S11	fine speckled and multiple nuclear dots (nuclear and cytoplasmic speckled)	OJ	PL-7 (weak)	─ *DBT-NUP210* gp210-sp100*	
S12	nuclear fine speckled, nucleolar staining and cytoplasmic fine speckled	─ *Th/To*	─ *Th/To, Ribosome*	─ *Th/To*	
S13	nuclear fine speckled and cytoplasmic dense fine speckled	─ *U1RNP*	─ U1RNP, SS-A, Ro52*	MDA5 (equivocal)* U1RN P70, U1RN PA, protein argonaute-2 (1, 3), Ro52*	
S14	nuclear fine speckled and cytoplasmic dense fine speckled	Jo-1 *Ro60, SS-B*	─ *Ro52, SS-B*	† *Ro60, SS-B, ABCE1*	
S15	Not performed	─	─		─
S16	Not performed	─	NXP2		NXP2
S17	Not performed	Ha	PL-7 Ro52*		─
S18	Not performed	SAE	─ SS-B, Ro52*		SUMO (SAE) SS-B, Ro (weak)
S19	nuclear fine speckled	─		─	─
S20	negative	─		─	─
S21	cytoplasmic dense fine speckled	Zo		Zo	Zo (weak)
S22	nuclear fine speckled and cytoplasmic dense fine speckled	─		─	─
S23	cytoplasmic dense fine speckled	OJ		OJ	OJ

*Positive result obtained using a complementary assay (ELISA or dot immunoassay), rather than the IP-based assay; this includes specificities outside the detection scope of the IP method used.

Green: MSA specificity reported by the laboratory.

Tan: No MSA reported.

Blue (†): MSA specificity outside the reporting scope of the laboratory workflow for this study.

Grey: Sample not analysed by the laboratory; therefore, no result available.

**Table 3:** Laboratory-reported MSA and MAA specificities for samples analysed by two centres.

Sample	IIF	Bath	Tokyo	Leuven	Oklahoma
S24	Not performed	OJ, Jo-1	EJ		
S25	Not performed	TIF1γ	TIF1γ *Ribosome*		
S26	Not performed	TIF1γ	TIF1γ		
S27	Not performed	─	─		
S28	Not performed	─	─		
S29	Not performed	SAE *SS-B*	─ SS-B, Ro52*		
S30	homogeneous/chromosomes	─		─	
S31	Not performed	Ha			†
S32	Not performed	─			─ *PM-Scl*

*Positive result obtained using a complementary assay (ELISA or dot immunoassay), rather than the IP-based assay; this includes specificities outside the detection scope of the IP method used.

Green: MSA specificity reported by the laboratory.

Tan: No MSA reported.

Blue (†): MSA specificity outside the reporting scope of the laboratory workflow for this study.

Grey: Sample not analysed by the laboratory; therefore, no result available.

The validated detection scope of each laboratory’s IP-based workflow, together with the contribution of complementary assays, is summarised in **Table 4**, and should be considered when interpreting inter-laboratory differences in reported MSA specificity.

**Table 4:** Laboratory workflows, MSA detection scope and proportion of samples reported as MSA-positive.

Lab	IP-based method(s)	Validated MSA detection scope of IP-based method(s)	Additional detection via complementary assays	Total samples tested	Samples with ≥1 MSA*	% MSA Positive
Bath	• ^35^S-IP (K562)	Zo, OJ, PL-7, PL-12, EJ, Jo-1, Ha, TIF1γ, Mi-2, SAE, SRP, KS	ELISA (MDA5, NXP2)	32	15	47%
Tokyo	• RNA-IP (HeLa) • IP-WB (HeLa)	OJ, KS, PL-7, PL-12, EJ, Jo-1, TIF1γ, Mi-2, NXP2, SAE	ELISA (ARS, MDA5, TIF1γ, Mi-2, SRP, HMGCR, NXP2, SAE)	24	11	46%
Leuven	• IP-MS (HeLa)	Zo, OJ, KS, PL-7, PL-12, EJ, TIF1β, TIF1γ, Mi-2	D-Tek dot immunoassay (MDA5, NXP2, TIF1γ, Jo-1, PL-7, PL-12, EJ, SRP, Mi-2, Ro52, SAE1, SAE2)	20	7	35%
Oklahoma	• RNA-IP (HeLa) • ^35^S-IP (HeLa) • IP-WB (K562)	Zo, OJ, PL-7, PL-12, EJ, Jo-1, TIF1γ, Mi-2, SUMO (SAE), SRP, NXP2, MDA5		18	9	50%

*One sample (S24) demonstrated reactivity to more than one MSA (anti-OJ and anti-Jo-1). All other samples contained a single MSA only.

### 2.3 Scope of autoantibody analysis

Participating laboratories reported a range of autoantibody reactivities detected during routine diagnostic testing. The present analysis focused exclusively on MSAs. Other autoantibody reactivities, including myositis-associated autoantibodies (MAAs) were recorded in the tables but were not included in the primary analysis. Accordingly, samples described as MSA negative in this study refer specifically to the absence of a defined MSA and do not necessarily indicate the absence of other autoantibody reactivities.

In instances where laboratories reported unidentified bands (UIB) derived from either protein or RNP autoantigens, such as “tRNA-UIB”, or other non-specific findings without assignment of a defined antibody specificity, these results were classified as no defined MSA identified for the purposes of concordance analysis.

### 2.4 Outcome definitions

The primary outcome was inter-laboratory concordance of MSA specificity. Exact concordance was defined as all laboratories within a given overlap group reporting the same MSA specificity. Majority agreement was defined as >50% of laboratories within a group reporting the same MSA specificity. Samples demonstrating less than majority agreement were classified as discordant. Samples in which no laboratory reported a defined MSA were classified separately as concordant MSA-negative cases.

Interpretation of concordance also considered differences in the antigen detection scope of each laboratory’s methodology. Using current cell extract preparation protocols, none of the IP workflows included here are capable of reliably detecting all MSAs. Where an MSA was identified but not reported by one or more centres because that specificity was outside the detection or reporting scope of their workflow, the finding was interpreted as methodologically consistent rather than discordant. Overall concordance therefore comprised exact MSA agreement, concordant MSA-negative results, and instances of methodologically expected non-detection. Majority agreement cases were not included in overall concordance unless the remaining non-detection was methodologically expected.

### 2.5 Antibody-level agreement analysis

Antibody-level performance was evaluated using two complementary metrics. A reproducibility index was calculated for each specificity as the total number of laboratory-level positive calls divided by the number of unique samples in which that antibody was detected by at least one laboratory. This reflects the average number of laboratories identifying a given antibody per positive sample and therefore captures cross-laboratory detectability. In parallel, a concordance rate was calculated as the proportion of unique positive samples demonstrating exact agreement (all laboratories reporting the same MSA specificity). The concordance rate therefore reflects sample-level agreement for each MSA. Together, these measures distinguish between the breadth of antibody detection across laboratories and the degree of inter-laboratory agreement on specificity.

## 3 Results

### 3.1 Overview

Thirty-four samples were distributed from the University of Bath Serology Service to three collaborating reference laboratories (Tokyo, Leuven, and Oklahoma), with partial overlap in sample testing. As the primary aim was to compare IP-based workflows, samples for which only non-IP assay results were available (e.g. those analysed by dot immunoassay alone) were excluded from the primary IP-based concordance analyses. Consequently, 32 samples were included in the final analysis.

Of these, seven samples were analysed by all four laboratories (including Bath), sixteen by three laboratories, and nine by two laboratories (**Figure 1**). The proportion of samples reported as MSA-positive varied between laboratories, ranging from 35% (Leuven) to 50% (Oklahoma) (**Table 4**). IIF data were available from the Leuven laboratory only and are presented as descriptive findings. Non-MSA reactivities are reported in the tables but were not included in the primary analysis of this paper.

**Figure 1:**
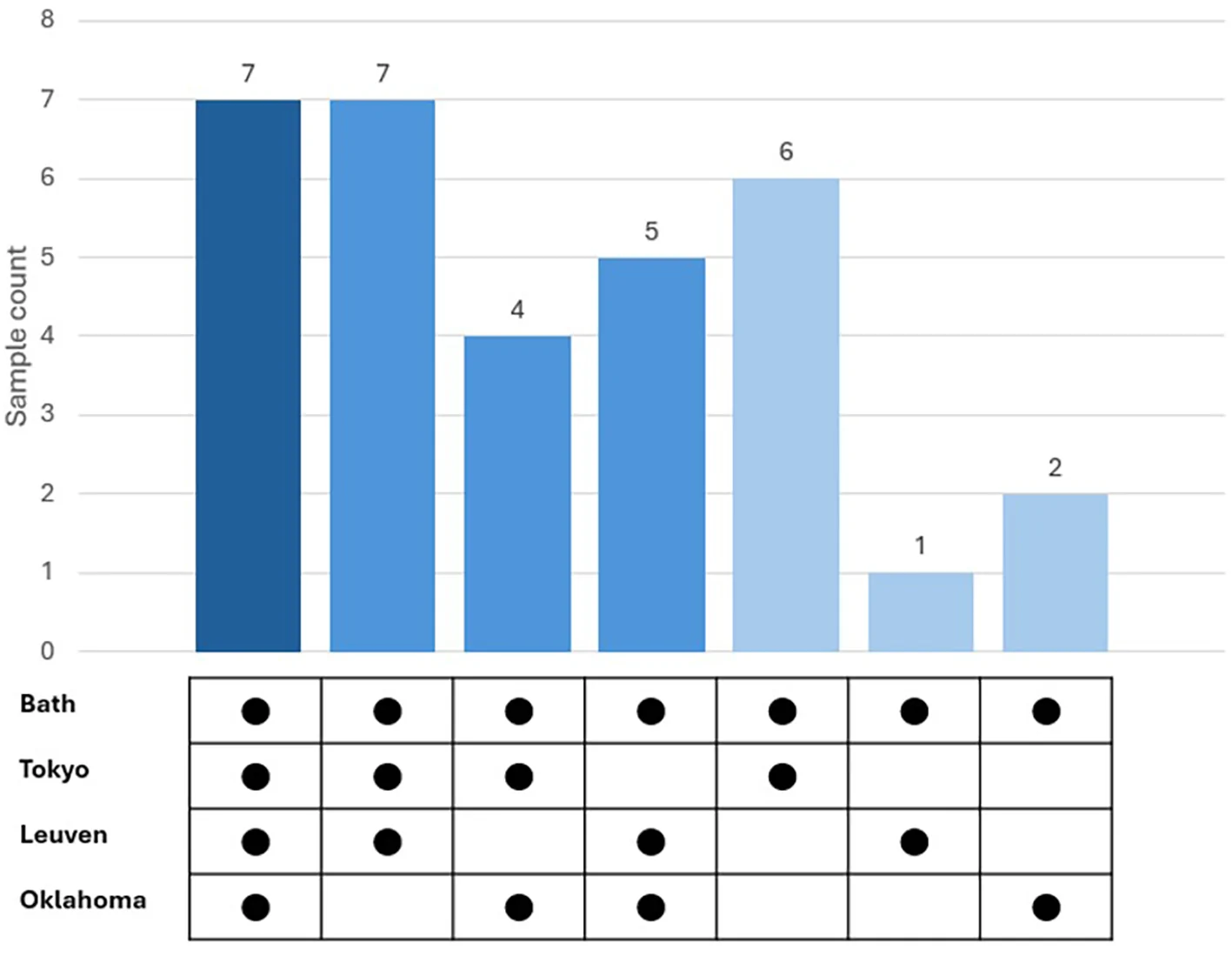
Sample overlap across laboratories. Bar colours indicate the number of laboratories represented in each overlap group: dark blue, four laboratories; medium blue, three laboratories; and light blue, two laboratories. Filled circles indicate the laboratories included in each group.

### 3.2 Inter-laboratory concordance of MSA specificity

Interpretation of inter-laboratory concordance must be considered in the context of differences in IP technique used, complementary and/or confirmatory assay selection and detection strategies across laboratories. Absence of a reported specificity at a given centre may therefore reflect methodological scope rather than absence of particular MSAs.

*Four-Laboratory Overlap (n = 7)*

Across the seven samples tested by all four laboratories, six demonstrated MSA positivity in at least one laboratory and were included in the primary specificity analysis (**Table 1**). Exact concordance for MSA specificity (all laboratories reporting the same MSA) was observed in 2/6 (33%) cases, involving TIF1γ and NXP2. Majority agreement was observed in 1/6 (17%) samples, in which three of four laboratories reported anti-Zo reactivity. This was reported by Bath, Leuven, and Oklahoma, but not by Tokyo, where anti-Zo was outside the reporting scope of the laboratory workflow. In this case, non-detection was considered methodologically expected for the purposes of overall concordance. In one sample, SRP reactivity was reported by Oklahoma using both RNA-IP and ^35^S-IP, and by Tokyo using ELISA, whereas the other laboratories did not report MSA positivity. In another sample, weak EJ reactivity was reported by Oklahoma but was not reported by the other centres. In a further sample, anti-HMGCR reactivity was identified by Tokyo using ELISA, while no MSAs were detected by the other laboratories, consistent with anti-HMGCR falling outside the detection/reporting scope of the IP-based workflows used by Bath, Leuven and Oklahoma. This finding was therefore considered methodologically expected for the purposes of overall concordance. One sample demonstrated complete concordance across all laboratories in the absence of MSA reactivity. Overall concordance was therefore observed in 5/7 (71%) samples.

*Three-Laboratory Overlap (n = 16)*

Of the sixteen samples tested by three laboratories, nine demonstrated MSA positivity in at least one laboratory (**Table 2**). Within this subset, exact concordance (3/3 laboratories reporting the same MSA) occurred in 3/9 (33%) samples (Zo, MDA5 and OJ), with majority agreement (2/3 laboratories reporting the same MSA) observed in 2/9 (22%) samples (NXP2 and SAE). The remaining 4/9 (44%) did not demonstrate either exact or majority concordance in MSA specificity. Seven samples demonstrated complete concordance in the absence of MSA reactivity across all three laboratories. Overall concordance was therefore observed in 10/16 (63%) samples.

*Pairwise Comparisons (n = 9)*

Nine samples were shared between two laboratories. Of these, five demonstrated MSA positivity in at least one laboratory and were included in the primary specificity analysis (**Table 3**). Within this subset, exact concordance (2/2 laboratories reporting the same MSA) was observed in 2/5 (40%) samples (TIF1γ), with discordant MSA specificity in 3/5 (60%). In one of these, anti-Ha was reported by Bath, while Oklahoma reported a tRNA-UIB but did not formally assign anti-Ha specificity, which was outside its reporting scope. This was therefore considered methodologically expected for the purposes of overall concordance. The remaining four samples demonstrated concordance in the absence of MSA reactivity. Overall concordance was therefore 7/9 (78%).

The results are summarised in **Table 5**. Overall concordance across overlap groups ranged from 63–78%. Agreement on MSA-negative status was high across centres, with multiple samples demonstrating concordant absence of MSA reactivity across all participating laboratories.

**Table 5:** Inter-laboratory concordance of MSA specificity across overlap groups.

Overlap group	Total samples	MSA-positive subset	MSA-positive concordance	Overall concordance*
4 Labs	7	6	3/6 (50%)	5/7 (71%)
3 Labs	16	9	5/9 (56%)	10/16 (63%)
2 Labs	9	5	2/5 (40%)	7/9 (78%)

*Overall concordance includes exact MSA concordance, concordant MSA-negative results and methodologically expected non-detection where the MSA was outside the detection/reporting scope of one or more participating laboratories.

### 3.3 Antibody-level detectability and agreement

At the antibody level, both detectability and cross-laboratory agreement varied by MSA specificity (**Table 6**). Anti-Zo, NXP2 and TIF1γ demonstrated the highest reproducibility indices (3.0, 3.0 and 2.7 respectively). TIF1γ showed complete concordance across all positive samples, whereas anti-NXP2 demonstrated full concordance in one sample and majority agreement in the remaining sample. Within the Tokyo workflow, all three anti-TIF1γ-positive samples were detected by IP-WB, while two were also positive by ELISA. In the latter NXP2 case, anti-NXP2 reactivity was detected using both IP-WB and ELISA at Tokyo and by IP-WB at Oklahoma, while UIB was observed on ^35^S-IP at Bath. Anti-Zo demonstrated concordant reporting among laboratories in which this specificity was within the reporting scope (Bath, Leuven and Oklahoma) but not Tokyo where anti-Zo reference sera are not included as standard.

**Table 6:** MSA detectability and inter-laboratory agreement metrics.

MSA	Total laboratory-level positive calls	Unique samples with ≥1 positive call	Reproducibility index	Full concordance	Bath	Tokyo	Leuven	Oklahoma
Zo	6	2	3.0	2/2 (100%)	2 (^35^S-IP)	*†*	2 (IP-MS)	2 (^35^S-IP)
NXP2	6	2	3.0	1/2 (50%)	1 (^35^S-IP with ELISA)	2 (IP-WB 2; ELISA 2)	1 (dot-blot)*	2 (IP-WB)
TIF1γ	8	3	2.7	3/3 (100%)	3 (^35^S-IP)	3 (IP-WB 3; ELISA 2)	1 (IP-MS)	1 (IP-WB)
MDA5	4	2	2.0	1/2 (50%)*	1 (^35^S-IP with ELISA)	1 (ELISA)*	2 (dot-blot)*	*‡*
SRP	2	1	2.0	0/1 (0%)	0	1 (ELISA)*	*†*	1 (RNA-IP, ^35^S-IP)
OJ	5	3	1.7	1/3 (33%)	3 (^35^S-IP)	0	1 (IP-MS)	1 (RNA-IP, ^35^S-IP)
SAE/SUMO	3	2	1.5	0/2 (0%)	2 (^35^S-IP)	0	*‡*	1 (^35^S-IP)
Jo-1	2	2	1.0	0/2 (0%)	2 (^35^S-IP)	0	*†*	*‡*
EJ	2	2	1.0	0/2 (0%)	0	1 (RNA-IP)	0	1 (RNA-IP, ^35^S-IP) (weak)
Ha	2	2	1.0	0/2 (0%)	2 (^35^S-IP)	*†*	*‡*	*†*
PL-7	2	2	1.0	0/2 (0%)	0	2 (RNA-IP) - one was weakly positive	0	0
HMGCR	1	1	1.0	0/1 (0%)	*†*	1 (ELISA)*	*†*	*†*

*Positive result derived from complementary assay rather than immunoprecipitation-based method.

†:Specificity outside the reporting scope of the laboratory workflow for this study.

‡:Not assessed, as no sample containing this specificity was available to this laboratory.

Anti-MDA5 showed intermediate reproducibility (index 2.0), with concordance observed in one of two samples. All four positive calls were derived from complementary assays (dot immunoassay and ELISA) with IP-based findings requiring confirmatory testing for definitive specificity assignment. Anti-SRP demonstrated similar reproducibility (2.0), with reactivity detected by two laboratories using different methodologies (ELISA and IP-based approaches), but without concordance across all centres. Anti-OJ demonstrated intermediate detectability (reproducibility index 1.7) with full concordance in one of three samples (33%). In the discordant samples, other laboratories detected alternative anti-synthetase specificities, including anti-EJ or PL-7.

In contrast, several specificities including anti-PL-7, Jo-1, EJ, Ha, and HMGCR were identified by only a single laboratory per positive sample within this dataset, resulting in reproducibility indices of 1.0 and no instances of cross-laboratory concordance. Notably, anti-HMGCR reactivity was detected only by ELISA in this study and not by IP-based assays. In several instances, laboratories detected anti-synthetase-associated reactivity but differed in the specificity assigned (e.g. anti-PL-7 versus OJ), and tRNA-UIBs were also reported. In the case of anti-Ha reactivity identified by Bath in two samples, Oklahoma observed banding patterns on both ^35^S-IP and RNA-IP that were suggestive of anti-Ha reactivity, based on similarity to previously characterised reference patterns. However, anti-Ha specificity was not formally assigned, as samples were not analysed alongside a confirmed control, and therefore did not meet the laboratory’s criteria for definitive reporting.

Where available, HEp-2 IIF patterns, reported only by Leuven, showed relatively good correlation with reported antibody specificities, including MAAs and other non-MSAs.

### 3.4 Laboratory-level detection patterns

Inspection of laboratory-level results revealed patterns associated with differences in IP workflows (**Table 6**). Anti-Zo reactivity was detected by laboratories employing protein-based IP approaches (Bath and Oklahoma) and IP-MS (Leuven), but not by the RNA-IP/IP-WB workflow used in Tokyo. Conversely, anti-PL-7 was reported only by RNA-IP (Tokyo); the relevant sample was not distributed to Oklahoma, precluding comparison with the second centre employing RNA-IP. Antibodies including anti-TIF1γ and NXP2 were detected across multiple methodological approaches. Anti-MDA5 detection varied between centres depending on the complementary assays used. In Bath, a 140-kDa band was detected by ^35^S-IP, with further in-house ELISA testing being required to distinguish anti-MDA5 from anti-NXP2 reactivity as both autoantigens have similar molecular weights. The current Leuven IP-MS workflow does not allow for detection of anti-MDA5, which necessitated complementary dot immunoassays. Similarly, because MDA5 is a protein antigen rather than part of a stable RNP complex, it cannot be detected by the RNA-IP-based testing employed by Tokyo. Anti-MDA5 specificity was therefore confirmed using a commercial ELISA. The two samples in which anti-MDA5 reactivity was reported were not distributed to Oklahoma, precluding assessment of detectability at that centre and limiting cross-method comparison for this specificity.

## 4 Discussion

### 4.1 Key findings

In this inter-laboratory comparison, variability in the detection and attribution of MSAs was observed across expert centres employing IP-based workflows. Although overall concordance was substantial across overlap groups, differences in MSA detection and specificity assignment occurred in several cases. These findings highlight that IP, while widely regarded as a reference approach for MSA detection, does not represent a single uniform assay consistently producing identical results.

IP encompasses a group of related but distinct laboratory techniques, all of which share a crucial IP step. The particular IP technique selected, and its methodological details, depend upon, and are tailored to, local laboratory infrastructure, expertise and available resources. The patterns observed in this study suggest that differences in locally implemented IP workflows, as well as inherent differences between the techniques themselves (such as RNA-IP only being able to detect RNP antigens), can influence which MSA specificities are detected and how results are interpreted. Importantly, when considered within the expected detection scope of each laboratory’s methodology, agreement between laboratories was generally high.

Agreement on the absence of MSA reactivity was also high across centres, with multiple samples demonstrating concordant MSA-negative results. However, a notable proportion of samples were reported as MSA-positive by only a single laboratory, highlighting that discrepancies in MSA detection remain an important source of inter-laboratory variability. While laboratories were often aligned in distinguishing MSA positive from negative samples, differences in detection and reporting thresholds can lead to discordant classification in a subset of cases.

Although the primary analysis focused on MSAs, several samples demonstrated additional MAAs or other non-myositis autoantibody reactivities. Inclusion of these findings provided important context for interpretation of HEp-2 IIF patterns and highlighted the broader autoantibody profiles identified through integrated laboratory workflows. It should be noted that a proportion of the concordant MSA-negative samples reported reactivities for non-myositis autoantibodies which were not always concordant between centres. Variation in the use of complementary assays across centres contributed to these differences, as certain specificities were identified only through non-IP methods. For example, anti-HMGCR reactivity was detected exclusively by ELISA, and some anti-MDA5 reactivity was identified using dot immunoassay, reflecting the influence of complementary assay selection on apparent inter-laboratory agreement.

Reliance on a single IP assay for MSA reporting is rarely performed in isolation since, using current protocols, it is not possible to confidently assess the status of all MSAs. Instead, MSA specificity is typically assigned through integration of multiple complementary methods, including line or dot immunoassay, ELISA, or other confirmatory and/or complementary tests. The final MSA result therefore reflects expert interpretation of combined laboratory findings rather than the output of an individual assay.

Lastly, samples included in this phase of the study were intentionally selected as challenging cases, including rare autoantibody specificities and samples with previously discordant results across commercial testing platforms. As such, the findings may represent a “worst-case” scenario for inter-laboratory comparison rather than typical routine diagnostic samples.

### 4.2 Methodological drivers of antibody detection

The variability observed between laboratories appears to be largely attributable to methodological differences between IP-based workflows. These include variation in antigen source, solid-phase matrix used for autoantibody immobilisation, detection strategy, and confirmatory testing approaches. Such differences can influence both the detectability of specific MSAs and the assignment of MSA specificity. In addition, it was evident that confirmatory and/or complementary assays often played an essential part in the overall determination of certain MSA specificities, particularly for anti-MDA5, NXP2 and HMGCR.

Within individual laboratory workflows, where the same MSA specificity was assessed by more than one method, findings were not always concordant. For example, among the three anti-TIF1γ positive samples reported by Tokyo, all three were detected by IP-WB, whereas only two were positive by ELISA. The same sample with discordant IP-WB and ELISA findings was independently identified as anti-TIF1γ by ^35^S-IP at Bath. Although this study was not designed to compare the analytical performance of individual assays, this finding highlights that different methods within a laboratory workflow may occasionally yield discrepant results, supporting the selective use of complementary or confirmatory testing where specificity assignment remains uncertain, rather than reliance on results of a single method in isolation.

Detection of the rarer anti-ARS antibodies in particular was often technically challenging, with differences being observed in their attribution. In several cases, laboratories detected evidence of an anti-ARS-associated immunoprecipitate but assigned different specificities, or reported an UIB, reflecting the challenges of distinguishing closely related ARS targets across different analytical approaches or, in the case of RNA-IP, the difficulty of identifying the particular RNA detected on a Urea-PAGE gel (even in the presence of known reference samples), when they often have very similar sizes. In contrast, the polypeptide components of ARS have a variety of molecular weights readily distinguished on SDS-PAGE gels.

The observed patterns were broadly consistent with differences between IP workflows. Within this sample cohort, ^35^S-IP approaches (Bath, Oklahoma) identified a broad range of MSAs (including anti-Zo, NXP2, TIF1γ, OJ, MDA5 (with ELISA confirmation), SAE, Jo-1, and Ha), whereas RNA-IP (Tokyo, Oklahoma) is only capable of detecting anti-synthetases and other RNPs.

Interpretation of IP results frequently relies on comparison with reference sera to assign antibody specificity. Techniques such as IP-MS and IP-WB can provide direct antigen identification, but IP workflows which rely on ^35^S-IP or RNA-IP remain dependent on recognition of characteristic banding patterns of protein-derived polypeptides or RNA patterns respectively. While RNA-IP can detect immunoprecipitated tRNA species associated with multiple ARS, assignment of specific anti-ARS antibodies relies on comparisons with tRNAs immunoprecipitated by established reference sera being run alongside. In the Tokyo workflow, reference sera were available for anti-Jo-1, PL-7, PL-12, EJ, OJ and KS, but not for anti-Zo and Ha, therefore specificity was not assigned to the latter two MSAs in the analysed samples. Interestingly, anti-HMGCR, first identified by ^35^S-IP in 2010 through the screening of sera from patients with IMNM (19), was only detected by ELISA (Tokyo) and not by IP-based methods in this study. A subsequent paper described that the addition of mevinolin, a statin that upregulates HMGCR expression, enhanced detection of characteristic 100/200 kDa bands and increased IP signal, although this approach is not routinely employed (20). However, in the ^35^S-IP protocol used, RIPA buffer was necessary to release HMGCR, a highly embedded integral membrane protein of the ER which is not solubilised by the non-ionic detergent buffers used for the ^35^S-IP methods described in the present study (21). Meanwhile, commercial websites indicate that the HMGCR used for ELISA and line/dot immunoassays is often of recombinant origin.

IP-MS (Leuven) detected several protein targets including anti-Zo, TIF1γ, and OJ. Detection of anti-MDA5 and anti-NXP2 is outside the scope of the current Leuven IP-MS workflow. The low sensitivity of the current IP-MS method for detection of these antigens likely reflects low antigen abundance in the cell extract used for this workflow (22–26). Although anti-Jo-1 and SRP are represented on the complementary D-Tek dot immunoassay, this assay was not performed on the relevant samples in this study, precluding assessment of their detection by this component of the Leuven workflow. In addition, selection of the nuclear or cytoplasmic extract for IP-MS was guided by the HEp-2 IIF pattern rather than both extracts being routinely analysed, introducing another potential source of variability. As interpretation of IIF patterns has an element of observer dependence, particularly where staining is weak or atypical, this may influence extract selection and consequently the opportunity to detect antibodies targeting antigens predominantly represented in the alternative extract. Samples with reported SAE and Ha reactivity were not distributed to Leuven, precluding assessment of detectability for these specificities.

These findings highlight that differences in antigen source and availability within assay preparations, criteria used to assign specificity (including availability of reference sera), and confirmatory testing may influence antibody identification. Inter-laboratory variation in MSA detection may therefore reflect differences in methodological implementation and interpretation rather than the absence of MSAs or inherent limitations of IP technology.

### 4.3 Clinical and research implications

The observations from this study have several implications for both clinical practice and research. In both settings, results generated by expert laboratories performing IP-based testing are often considered definitive reference findings. However, the present data demonstrate that differences in IP technique used and laboratory-specific IP workflows can influence which MSAs are detected and how specificity is assigned. Awareness of methodological differences and assay-specific limitations may assist clinicians when interpreting laboratory results, particularly when antibody findings appear discordant with clinical features. In this cohort, discordant results occurred predominantly among anti-ARS antibodies, where the associated clinical phenotype is broadly comparable across different anti-ARS specificities. Differences in attribution between these specificities (e.g., EJ versus OJ) may therefore have less clinical impact compared with discordance between distinct MSA subtypes.

As MSAs are increasingly used to define clinical subsets of IIM and to stratify patients within research studies, awareness of methodological variation and its influence on MSA detection and identification is important for interpreting and comparing findings across patient cohorts and studies.

Translating these findings into practice, harmonisation efforts may benefit from greater transparency and more standardised reporting of IP-based methodologies. Studies and laboratories should specify the form of IP used, the range of MSA specificities that can be reliably detected using the IP method alone, and any recognised method-specific limitations. Where downstream identification or complementary assays are used to confirm or contribute to specificity assignment, this should also be explicitly stated. Such reporting would facilitate more appropriate interpretation and comparison of results between studies. These principles could provide a basis for future consensus recommendations developed through international collaborative groups, such as the International Myositis Assessment and Clinical Studies Group (IMACS) and the European Autoimmunity Standardisation Initiative (EASI).

### 4.4 Strengths

To our knowledge, this is the first study directly comparing inter-laboratory MSA results using different IP-based diagnostic workflows across multiple expert reference centres. The analysis reflects real-world diagnostic practice, in which laboratories employ established but locally developed workflows and integrate results from multiple assays to assign MSA specificity.

### 4.5 Limitations

Several limitations should be considered. Not all samples were analysed by all laboratories, resulting in only partial overlap between centres. The small number of positive samples for several MSAs limited conclusions regarding method-specific detectability for those antibodies. In particular, the rarer anti-ARS, anti-SRP and anti-HMGCR antibodies were represented by very small numbers of positive samples, limiting the statistical robustness of antibody-level reproducibility analysis. Findings should therefore be interpreted as descriptive observations within this selected cohort rather than precise estimates of inter-laboratory reproducibility. Samples included in this study were intentionally selected as challenging cases, including rare autoantibody specificities and samples with previously discordant IP versus commercial LIA results. The findings may not reflect the frequency of inter-laboratory discordance in routine diagnostic practice. While this approach enabled evaluation of methodological differences, the cohort may represent a more challenging scenario than routine diagnostic practice and may have amplified the apparent level of variability. In addition, matched clinical data were not available for samples, limiting assessment of the clinical significance of discordant MSA findings. Agreement in routine clinical cohorts may be higher for common, strongly reactive MSAs that are well represented across assay platforms, whereas rare, weak or method-dependent specificities may be more susceptible to discordant detection or specificity assignment. Future studies should include unselected diagnostic samples with matched clinical information to determine the extent to which these findings apply to routine clinical practice.

### 4.6 Conclusions

This inter-laboratory comparison demonstrates that both the specific IP techniques utilised and IP-based workflows for the detection of MSAs can vary across expert centres despite shared methodological principles. Regarding IP techniques, the differences observed may primarily reflect variation in antigen source, detection modality, the nature of confirmatory assays, and interpretive frameworks within locally implemented laboratory workflows.

These findings highlight that IP should be viewed not as a single uniform assay but as a family of related techniques embedded within different expertly interpreted diagnostic workflows. Recognising this methodological diversity including specific assay limitations is important when interpreting so-called “reference laboratory” results and when comparing MSA data across clinical studies and cohorts. Efforts to harmonise MSA testing may benefit from acknowledging this diversity, rather than assuming complete standardisation of IP-based assays. Greater transparency in laboratory methodologies and reporting frameworks may represent an important step toward harmonising MSA testing across centres. This may also facilitate more consistent comparison of MSA findings across laboratories and research studies.

## Data Availability

The original contributions presented in the study are included in the article/supplementary material. Further inquiries can be directed to the corresponding author.
